# RETRACTED: Chen et al. Rosmarinic Acid Attenuates the Lipopolysaccharide-Provoked Inflammatory Response of Vascular Smooth Muscle Cell via Inhibition of MAPK/NF-κB Cascade. *Pharmaceuticals* 2022, *15*, 437

**DOI:** 10.3390/ph19010046

**Published:** 2025-12-25

**Authors:** Ching-Pei Chen, You-Cian Lin, Yu-Hui Peng, Han-Min Chen, Jiun-Tsai Lin, Shao-Hsuan Kao

**Affiliations:** 1 Division of Cardiology, Department of Internal Medicine, Changhua Christian Hospital, Changhua 50006, Taiwan; 72809@cch.org.tw; 2 Cardiovascular Division, Surgical Department, China Medical University Hospital, Taichung 404332, Taiwan; lhh0203@gmail.com; 3 School of Medicine, China Medical University, Taichung 404332, Taiwan; 4 Institute of Medicine, College of Medicine, Chung Shan Medical University, Taichung 402306, Taiwan; me5124ya@gmail.com; 5 Institute of Applied Science and Engineering, Catholic Fu Jen University, New Taipei 242048, Taiwan; 056489@mail.fju.edu.tw; 6 Energenesis Biomedical Co., Ltd., Taipei 114694, Taiwan; jt@energenesis-biomedical.com; 7 Department of Medical Research, Chung Shan Medical University Hospital, Taichung 402306, Taiwan

The journal retracts the article “Rosmarinic Acid Attenuates the Lipopolysaccharide-Provoked Inflammatory Response of Vascular Smooth Muscle Cell via Inhibition of MAPK/NF-κB Cascade” [1] cited above.

Following publication, concerns were brought to the attention of the Editorial Office regarding the integrity of images presented in this publication [1].

Adhering to standard procedure, an investigation was conducted by the Editorial Office and the Editorial Board, which identified indications of inappropriate editing and partial duplication between Figure 3B presented in this article [1] and Figure 5A of an earlier publication [2], produced by a similar research group and presenting different experimental conditions. While the authors collaborated during this process, raw materials meeting the journal’s requirements for original images (https://www.mdpi.com/journal/pharmaceuticals/instructions#oriimages) could not be provided for Editorial Board evaluation. Consequently, the Editorial Board has lost confidence in the reliability of the findings and has decided to retract this publication, as per MDPI’s retraction policy (https://www.mdpi.com/ethics#_bookmark30).

This retraction was approved by the Editor-in-Chief of the journal *Pharmaceuticals*.

The authors did not provide a comment on this decision.
